# Low serum Maresin-1 levels are associated with non-alcoholic fatty liver disease: a cross-sectional study

**DOI:** 10.1186/s12944-021-01518-5

**Published:** 2021-08-30

**Authors:** Xia Fang, Hongya Wang, Ting Ye, Xiaolan Fu, Xiaozhen Tan, Yan Zeng, Jiahao Fan, Yong Xu

**Affiliations:** 1 Department of Endocrinology and Metabolism, the Affiliated Hospital of Southwest Medical University, 25 Taiping Street, Jiangyang District, Luzhou, 646000 Sichuan China; 2 Cardiovascular and Metabolic Diseases Key Laboratory of Luzhou, Luzhou, 646000 Sichuan China; 3 Sichuan Clinical Research Center for Nephropathy, Luzhou, 646000 Sichuan China; 4 Department of Laboratory Medicine, the Affiliated Hospital of Southwest Medical University, Luzhou, 646000 Sichuan China; 5 Department of Respiratory Medicine, Yongchuan Hospital of Traditional Chinese Medicine Affiliated to Chongqing Medical University, Chongqing, 402160 China; 6 Department of Gastroenterology, the Affiliated Hospital of Southwest Medical University, 25 Taiping Street, Jiangyang District, Luzhou, 646000 Sichuan China

**Keywords:** Maresin-1, Non-alcoholic fatty liver disease, Cross-sectional study, Metabolic diseases, Specialized pro-resolving mediators, Anti-inflammatory cytokines

## Abstract

**Background:**

Maresin-1 (MaR1) is an anti-inflammatory pro-resolving mediator and is considered a potential regulator of metabolic diseases. Non-alcoholic fatty liver disease (NAFLD) is a very common metabolic liver disease. However, little information is available on the relationship between MaR1 and NAFLD in humans. Therefore, the study explored the association between serum MaR1 levels and NAFLD.

**Methods:**

A cross-sectional study was conducted in 240 Chinese people, including 116 non-NAFLD subjects and 124 NAFLD patients. Serum MaR1 levels were determined by enzyme-linked immunosorbent assay (ELISA). The association between MaR1 and NAFLD was assessed.

**Results:**

Circulating MaR1 levels in NAFLD patients were markedly lower than those in non-NAFLD subjects (63.63 [59.87–73.93] vs 73.11 [65.12–84.50] pg/mL, *P* = 0.000). The percentages of patients with NAFLD gradually decreased with the increase of MaR1 quartiles (*P* < 0.001). Furthermore, serum MaR1 levels were positively associated with aspartate aminotransferase/alanine aminotransferase (AST/ALT), albumin, the albumin-globulin-ratio, and high-density lipoprotein cholesterol (HDL-C) (all *P* < 0.05) and negatively associated with body mass index (BMI), waist circumference, hip circumference, the waist-to-hip ratio, ALT, gamma-glutamyl transpeptidase (GGT), uric acid, triglyceride (TG), and fasting blood glucose (FBG) (all *P* < 0.05) after adjusting for sex and age. Binary logistic regression analysis revealed that serum MaR1 levels were significantly associated with NAFLD.

**Conclusions:**

Circulating MaR1 levels were decreased in patients with NAFLD, and a negative correlation was identified between NAFLD and serum MaR1 concentrations. Decreased MaR1 might be involved in the development of NAFLD.

## Background

Non-alcoholic fatty liver disease (NAFLD), which is the most common metabolic liver disease and one of the most common causes of chronic liver disease, is characterized by ectopic deposition of fat in hepatocytes without secondary causes of liver fat accumulation (e.g., excess alcoholic consumption, medication, viral infection) [1]. NAFLD has a growing impact on world health. The global prevalence of NAFLD is estimated to be as high as 25% [2]. Given the tremendous changes in lifestyles in the past 20 years, the prevalence of NAFLD in China has reached as high as 22.4%, which is equivalent to that in the United States (24.13%), Europe (23.71%) and Japan (25%) [2–6]. This rising prevalence of NAFLD will inflict an increasing economic burden and will be accompanied by an increasing number of patients with cirrhosis, liver transplantation or/and hepatocellular carcinoma (HCC) [7–10]. However, NAFLD is a heterogeneous disease with very different clinical manifestations and different rates of progression among individuals. Some patients are asymptomatic and only found to have simple non-alcoholic fatty liver (NAFL) during physical examination, while others present with severe nonalcoholic steatohepatitis (NASH), liver cirrhosis, or even HCC. Multiple factors have been identified as pathogenic drivers of NAFLD. Among these factors, an imbalance between pro- and anti-inflammatory systems is considered an important mechanism. NAFLD is regarded as a disorder featuring deficiencies in anti-inflammatory cytokines and bioactive lipids [11, 12]. Therefore, novel anti-inflammatory molecules must be identified to better investigate NAFLD progression and to screen high-risk groups who may be particularly susceptible to NAFLD.

Maresins constitute the third-largest family of specialized pro-resolving mediators (SPMs) derived from docosahexaenoic acid (DHA), which are mainly biosynthesized in M2 macrophages [13, 14]. Maresin-1 (MaR1) is the first discovered member of this family. Currently, increasing evidence shows that MaR1 plays vital roles in anti-inflammatory actions and metabolic diseases. In ob/ob and diet-induced obese mice, MaR1 improves insulin sensitivity and reverses adipose tissue dysfunction and inflammation [15]. MaR1 ameliorates liver steatosis by inhibiting endoplasmic reticulum stress and lipogenic enzymes and inducing autophagy via the AMP-activated protein kinase (AMPK) pathway in high fat diet (HFD)-fed mice [16, 17]. Recently, Han et al. showed that MaR1 protected mice from HFD-induced NASH by activating M2 polarization of liver macrophages in a retinoic acid-related orphan receptor α (RORα) dependent manner [18]. All these studies indicate that MaR1 is closely related to metabolic diseases, especially NAFLD in cells and animal models. However, the relationship between serum MaR1 levels and NAFLD in humans is still unclear.

In the present cross-sectional study, subjects with NAFLD were recruited to investigate alterations in circulating MaR1 and to explore the potential relationship between MaR1 levels and NAFLD.

## Methods

### Study population and design

non-NAFLD subjects and NAFLD patients were recruited from the Physical Examination Center of the Affiliated Hospital of Southwest Medical University. All ultrasonographic examinations were performed using a Doppler sonography system (ACUSON Sequoia, SIEMENS, Germany) to diagnose NAFLD by the same group of experienced sonographers following standardized procedures. NAFLD was diagnosed based on the presence of hepatic fat accumulation according to the criteria issued by the Chinese Liver Disease Association [19]. All of the following subjects were excluded: 1) excess alcohol consumption [20] or alcoholic liver disease, genetic liver diseases, drug- or toxin-induced liver diseases, biliary obstructive diseases, viral or autoimmune hepatitis, renal disease, human immunodeficiency virus (HIV) infection, cancer, acute or chronic inflammatory disease, cardiovascular or cerebral vascular disease, or pregnancy or breastfeeding; 2) systemic corticosteroid treatment, anti-inflammatory therapy, hypoglycaemic or lipid-lowering therapy, and antihypertensive treatment.

After screening, 124 patients with NAFLD and 116 subjects without NAFLD aged between 22 and 71 years participated in the study. All participants were categorized into quartiles based on their serum MaR1 concentration: quartile 1, MaR1 < 62.13 pg/mL; quartile 2, 62.13 pg/mL ≤ MaR1 < 68.71 pg/mL; quartile 3, 68.71 pg/mL ≤ MaR1 ≤ 77.79 pg/mL; and quartile 4, MaR1 > 77.79 pg/mL.

All experimental protocols followed Declaration of Helsinki and were endorsed by the Clinical Trial Ethics Committee of the Affiliated Hospital of Southwest Medical University (permission no. KY2021086). All participants have signed informed consent forms.

### Anthropometric and biochemical measurements

In the present study, all anthropometric measurements were performed before breakfast, and all participants wore light clothing and no footwear. After overnight fasting (approximately 10–12 h), anthropometric parameters were measured by a designated specialist nurse. After resting for at least 5 min, blood pressure (BP) was measured using a medical automatic electronic sphygmomanometer (HBP-9020, OMRON Corp., Kyoto, Japan). Height and body weight (BW) were measured by using an ultrasonic analyser (SK-V7, Shenzhen, China). Body mass index (BMI) was calculated as BW (kg)/height (m^2^). Waist circumferences (WC, the midpoint between the ilium and the lowest margin of the ribs) and hip circumference (HC, the maximum circumference of the hips) were measured with a cloth measuring tape. The waist-to-hip ratio (WHR) was calculated as WC (cm)/HC (cm).

After overnight fasting, serum samples were obtained from all subjects in the morning of the day of the ultrasound examination. Serum samples were collected and stored at − 80 °C until analysis. Alanine aminotransferase (ALT), aspartate aminotransferase (AST), gamma-glutamyl transpeptidase (GGT), alkaline phosphatase (ALP), total protein (TP), albumin (ALB), globulin (GLO), urea nitrogen (Urea), uric acid (UA), creatinine (Crea), total cholesterol (TC), triglycerides (TG), high-density lipoprotein cholesterol (HDL-C), low-density lipoprotein cholesterol (LDL-C), Fasting blood glucose (FBG), and homocysteine (HCY) were detected by a biochemical autoanalyser (ADVIA2400, SIEMENS, Germany). Peripheral white blood cell (WBC) and neutrophil (NEU) counts were measured by a hematology analyser (Mindray BC-6800, Shenzhen, China).

### Serum MaR1 measurement

Serum MaR1 levels were quantified using commercial enzyme-linked immunosorbent assay (ELISA) kits (Human ELISA kit, Senbeijia, NanJing, China) according to the manufacturer’s protocol. Serum samples were diluted 5-fold before the assay. The intra- and inter-assay variations were < 9% and < 11%, respectively. The detectable range of the kit was 5 pg/mL-160 pg/mL.

### Statistical analysis

SPSS 22.0 software (SPSS Inc., IL., USA) and GraphPad Prism 8.0 were used for all statistical analysis and graphics. Data were expressed as the number (percentage) for categorical variables or as the mean ± the standard deviation (SD) or medians [25th, 75th percentiles] for continuous variables, unless otherwise specified. Differences between the two groups were examined using Student’s t-test or the Mann-Whitney U-test for continuous variables. Differences among more than two groups were analysed with one-way analysis of variance (ANOVA) or the Kruskal-Wallis test for continuous variables. Categorical variables were compared using the chi-square test. Correlations between serum MaR1 and other clinical parameters was determined by Pearson correlation or Spearman correlation test according to the distribution of the parameters. Partial correlation coefficients were used for age- and sex-adjusted data. Binary logistic regression analyses were used to analyse the association between serum MaR1 levels and NAFLD. A *P* value < 0.05 was defined as statistical significance.

## Results

### General characteristics and serum MaR1 levels of the study participants

A total of 116 non-NAFLD subjects and 124 NAFLD patients were enrolled in this study. The average age of the subjects was 43.44 ± 11.36 years, including 134 males (55.8%) and 106 females (44.2%). Table 1 summarizes the general anthropometric, biochemical and clinical parameters and circulating MaR1 levels of the subjects. Compared to non-NAFLD subjects, NAFLD patients had higher BMI, BP, WC, HC, WHR, FBG, WBC, ALT, AST, GGT, ALP, Urea, UA, Crea, TC, TG, LDL-C, and HCY levels (all *P* < 0.05) and lower AST/ALT and HDL-C levels (both *P* < 0.001). The proportion of males was higher among subjects with NAFLD than among those without NAFLD (*P* = 0.029). There were no significant differences in age, neutrophil count, TP, ALB, GLO, and the albumin-globulin-ratio (A/G).

**Table 1:** Main clinical parameters and serum Maresin-1 levels in all participants.

Variables	non-NAFLD	NAFLD	*P*-value
Male/Female	57/59	77/47	0.029
Age (year)	41.50 (33.00-51.00)	45.50 (35.00-53.00)	0.286
BMI (kg/m2)	22.54 (21.20-24.20)	27.26 (25.48-29.49)	0.000
WC (cm)	79.68 ± 8.52	90.39 ± 7.92	0.000
HC (cm)	95.00 (91.00-98.00)	100.00 (96.00-104.75)	0.000
WHR	0.84 ± 0.06	0.89 ± 0.05	0.000
SBP (mmHg)	118.00 (110.00-124.00)	130.50 (119.00-138.00)	0.000
DBP (mmHg)	71.45 ± 9.21	77.29 ± 10.68	0.000
FBG (mmol/L)	4.95 (4.68-5.23)	5.32 (4.90-5.75)	0.000
WBC (*10^9/L)	6.11 ± 1.22	6.75 ± 1.35	0.000
NEU (*10^9/L)	3.65 ± 0.92	3.85 ± 0.95	0.099
ALT (U/L)	18.20 (13.30-27.20)	27.90 (21.25-42.05)	0.000
AST (U/L)	20.20 (17.30-26.05)	23.40 (19.43-27.50)	0.002
AST/ALT	1.15 (0.92-1.34)	0.83 (0.66-0.99)	0.000
TP (g/L)	73.00 ± 3.01	72.63 ± 2.93	0.328
ALB (g/L)	46.25 (45.00-47.63)	46.05 (45.03-47.80)	0.970
GLO (g/L)	26.62 ± 2.52	26.25 ± 2.59	0.260
A/G	1.76 (1.62-1.90)	1.77 (1.65-1.91)	0.501
GGT (U/L)	16.05 (13.10-26.85)	31.55 (20.58-48.60)	0.000
ALP (U/L)	67.40 (53.95-79.78)	74.05 (62.55-88.05)	0.003
Urea (mol/L)	4.70 (3.90-5.63)	5.13 (4.59-5.93)	0.004
UA (μmol/L)	320.00 (261.48-380.78)	370.15 (316.65-440.00)	0.000
Crea (μmol/L)	63.35 (53.80-71.40)	70.50 (55.75-77.23)	0.006
TC (mmol/L)	4.73 ± 0.77	4.95 ± 0.72	0.022
TG (mmol/L)	1.16 (0.84-1.67)	2.00 (1.42-2.67)	0.000
HDL-C (mmol/L)	1.35 (1.17-1.66)	1.08 (0.96-1.22)	0.000
LDL-C (mmol/L)	3.10 ± 0.83	3.42 ± 0.70	0.002
HCY (μmol/L)	9.55 (8.00-11.50)	10.65 (8.80-12.10)	0.009
Maresin-1 (pg/mL)	73.11 (65.12-84.50)	63.63 (59.87-73.93)	0.000

**Fig. 1:**
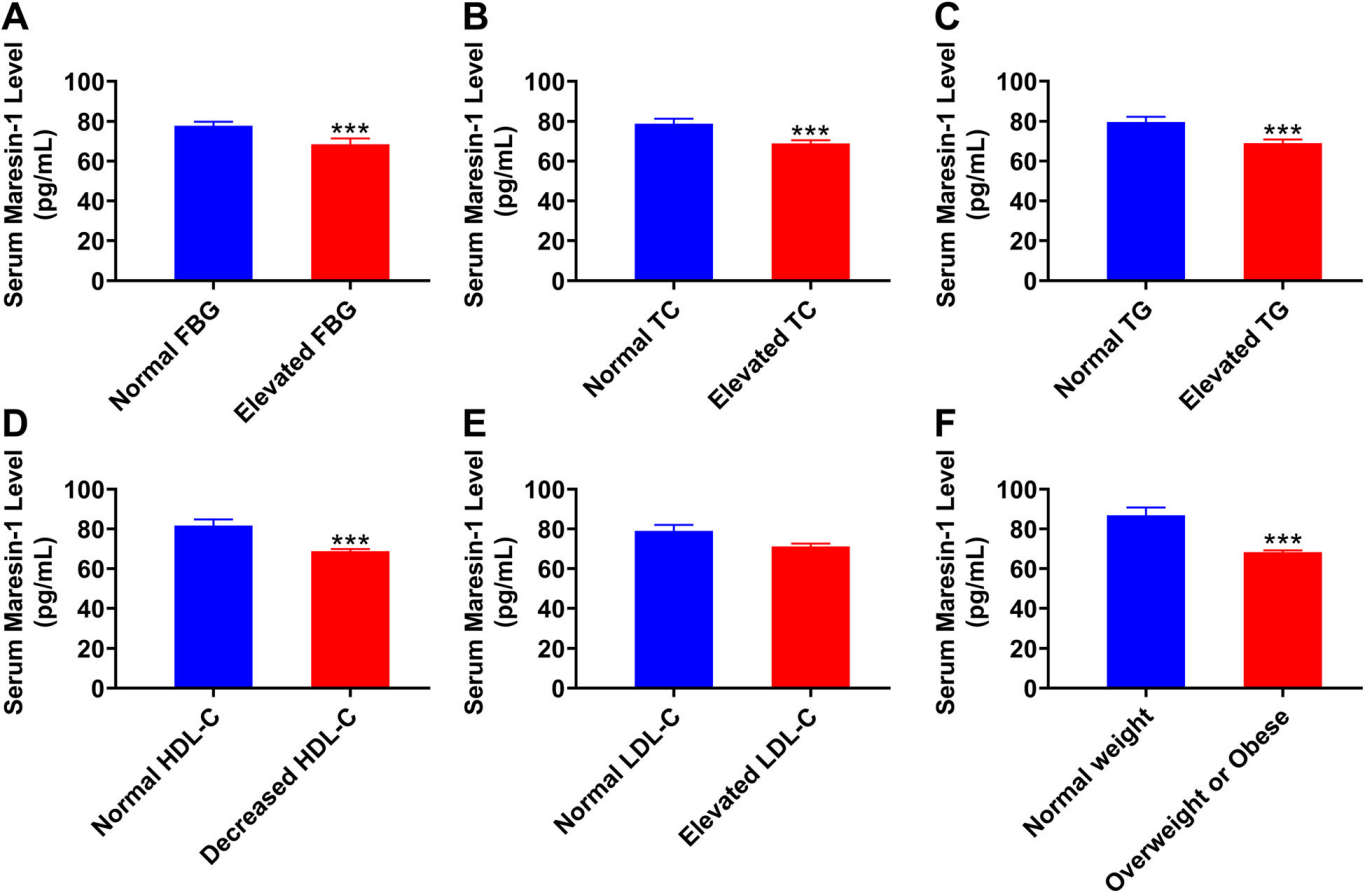
Serum MaR1 concentration in the study population under different metabolic conditions. **A** normal FBG (*n* = 177) versus elevated FBG (*n* = 63), (**B**) normal TC (*n* = 156) versus elevated TC (*n* = 84), (**C**) normal TG (*n* = 143) versus elevated TG levels (*n* = 97), (**D**) normal HDL-C (*n* = 123) versus decreased HDL-C levels (*n* = 117), (**E**) normal LDL-C (*n* = 128) versus elevated LDL-C (*n* = 112), and (**F**) normal weight (*n* = 92) versus with overweight and obesity (*n* = 148). *P < 0.05, **P < 0.01, ***P < 0.001 versus normal FBG, TC, TG, LDL-C, HDL-C levels or normal weight. The data are expressed as the means ± SEM

In all study subjects, serum MaR1 concentration ranged from 51.65–224.45 pg/mL. No significant difference in circulating MaR1 levels were found between men and women (66.99 [61.18–77.40] vs 71.52 [62.57–79.32], *P* = 0.078). In addition, to understand the serum levels of MaR1 under different metabolic conditions, all participants were divided into normal and abnormal groups according to the levels of FBG, lipids or BMI [21, 22]. As shown in Fig. 1, serum MaR1 levels were significantly lower in subjects with elevated FBG, TC and TG levels (all *P* < 0.001, Fig. 1A-C) and decreased HDL-C levels (*P* < 0.001; Fig. 1D) and in overweight or obese subjects (*P* < 0.001; Fig. 1F) than in the controls. No significant difference in serum MaR1 levels was found among subjects with different LDL-C levels (*P* = 0.2587; Fig. 1E). Importantly, serum MaR1 levels in NAFLD patients were significantly decreased compared with those in non-NAFLD subjects (*P* = 0.000, Table 1).

### Clinical and biochemical characteristics by quartiles of serum MaR1 in all study participants

**Fig. 2:**
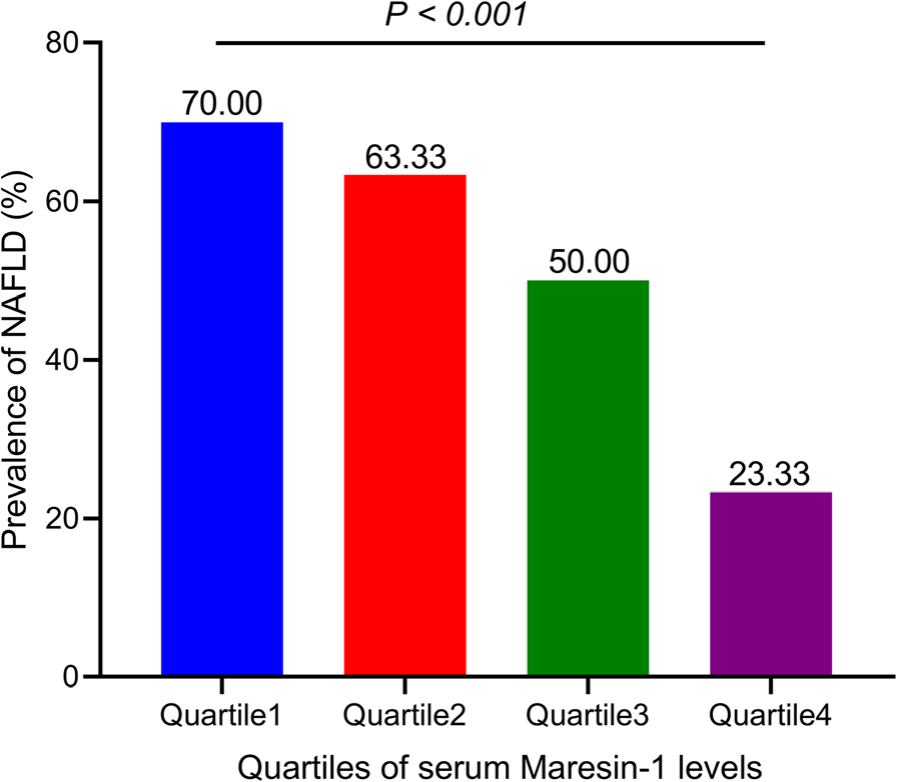
Prevalence of NAFLD by quartiles of circulating MaR1 in all participants

Table 2 shows general characteristics according to quartiles of serum MaR1 levels in all subjects. BMI, WC, HC, WHR, BP, FBG, ALT, AST, AST/ALT, GLO, A/G, GGT, ALP, Urea, TG, HDL-C, and MaR1 concentrations were significantly different between participants in different serum MaR1 quartiles (all *P* < 0.05). Subjects in the highest quartile of serum MaR1 concentration had lower BMI, WC, HC, WHR, BP, FBG, WBC, ALT, GLO, GGT, Urea, TC, TG, and LDL-C levels (all *P* < 0.05) and higher serum MaR1, AST/ALT, A/G, and HDL-C levels (all *P* < 0.01) than those in the lowest quartile of serum MaR1 concentration. As shown in Fig. 2, the prevalence of NAFLD rapidly decreased in tandem with increasing quartiles of serum MaR1 levels (*P* < 0.001).

**Table 2:** Clinical and biochemical characteristics by quartile of serum Maresin-1 level in all subjects.

Variables	Quartile 1	Quartile 2	Quartile 3	Quartile 4	*P*-value
	(~ < 62.13)	(62.13 ≤ - < 68.71)	(68.71 ≤ - < 77.79)	(~ ≥ 77.79)	
Sample Size	60	60	60	60	-
Male/Female	37/23	38/22	28/32	31/29	0.198
Age (year)	46.00 (40.25-52.75)	46.00 (34.00-54.00)	38.50 (31.00-51.75)	40.00 (31.25-49.75)	0.075
BMI (kg/m2)	26.13 (24.19-28.93)	26.27 (23.75-28.25)	25.23 (21.99-27.89)	23.06 (21.17-25.60) ***	0.000
WC (cm)	89.20 ± 9.73	87.13 ± 9.39	83.67 ± 9.29 **	80.85 ± 8.83 ***	0.000
HC (cm)	99.00 (94.25-104.00)	98.00 (93.00-102.00)	97.50 (93.25-103.00)	95.00 (92.00-99.00) *	0.017
WHR	0.90 (0.88-0.93)	0.89 (0.84-0.92)	0.85 (0.81-0.90) **	0.85 (0.80-0.88) ***	0.000
SBP (mmHg)	130.50 (118.00-137.00)	123.00 (115.00-134.75)	121.50 (111.00-134.75)	120.00 (112.00-129.50) *	0.038
DBP (mmHg)	76.93 ± 10.84	75.95 ± 10.51	73.78 ± 10.31	71.20 ± 9.15 **	0.012
FBG (mmol/L)	5.47 (4.86-5.81)	5.09 (4.83-5.68)	5.06 (4.73-5.38) *	4.98 (4.68-5.27) **	0.001
WBC (*10^9/L)	6.72 ± 1.45	6.59 ± 1.33	6.30 ± 1.17	6.17 ± 1.29 *	0.079
NEU (*10^9/L)	3.67 (3.08-4.47)	3.69 (3.07-4.58)	3.55 (3.11-4.12)	3.67 (2.99-4.43)	0.830
ALT (U/L)	26.70 (19.45-38.18)	27.40 (19.48-36.70)	20.90 (13.33-30.35) *	19.20 (13.98-27.88) **	0.000
AST (U/L)	23.20 (19.45-29.63)	23.30 (19.98-28.40)	20.15 (16.83-26.38)	20.15 (17.53-24.78)	0.010
AST/ALT	0.85(0.67-1.06)	0.88(0.67-1.14)	1.02(0.83-1.21) *	1.11(0.85-1.28) **	0.000
TP (g/L)	73.10 ± 2.88	73.01 ± 2.54	72.20 ± 3.37	72.95 ± 3.00	0.321
ALB (g/L)	45.95 (45.00-47.00)	45.85 (45.10-47.78)	45.90 (44.90-47.48)	47.25 (45.33-48.83)	0.058
GLO (g/L)	27.11 ± 3.05	26.64 ± 2.34	26.01 ± 2.44 *	25.97 ± 2.20 *	0.039
A/G	1.72 (1.55-1.83)	1.73 (1.60-1.88)	1.79 (1.66-1.91)	1.81 (1.73-1.94) *	0.009
GGT (U/L)	30.90 (19.23-47.33)	29.10 (18.03-42.53)	20.35 (14.68-31.40) *	15.40 (12.68-28.30) ***	0.000
ALP (U/L)	73.85 (56.45-84.73)	73.80 (62.43-84.70)	72.15 (61.23-87.53)	63.20 (52.40-79.13)	0.031
Urea (mol/L)	5.16 (4.83-5.96)	4.98 (4.40-5.90)	4.84 (3.97-5.77)	4.76 (3.96-5.41) *	0.027
UA (μmol/L)	356.55 (296.10-418.60)	362.35 (314.58-434.23)	332.80 (283.48-400.93)	318.55 (269.15-415.45)	0.160
Crea (μmol/L)	68.70 (55.95-75.25)	66.70 (54.38-74.38)	65.40 (52.63-73.78)	65.20 (54.93-74.95)	0.735
TC (mmol/L)	5.00 ± 0.80	4.92 ± 0.75	4.74 ± 0.77	4.71 ± 0.67 *	0.098
TG (mmol/L)	1.81 (1.28-2.65)	1.79 (1.23-2.63)	1.37 (0.91-1.82) **	1.21 (0.90-1.69) ***	0.000
HDL-C (mmol/L)	1.10 (0.99-1.34)	1.11 (0.96-1.24)	1.24 (1.08-1.46)	1.33 (1.17-1.63) **	0.000
LDL-C (mmol/L)	3.42 ± 0.79	3.32 ± 0.82	3.24 ± 0.77	3.07 ± 0.72 *	0.093
HCY (μmol/L)	10.05 (8.40-11.78)	10.10 (8.60-12.20)	10.10 (8.70-11.90)	10.15 (7.63-12.10)	0.866

Continuous variables are mean ± standard deviation or medians (25^th^, 75^th^ percentiles). *P < 0.05, **P < 0.01, ***P < 0.001 versus quartile 1 group.

### Association of Serum MaR1 levels with clinical parameters in the study population

Next, correlation analysis was performed to investigate associations between serum MaR1 levels and other clinical parameters. As shown in Table 3, in all study population, serum MaR1 concentrations were positively associated with AST/ALT, ALB, A/G, and HDL-C (all *P* < 0.05) and negatively associated with age, BMI, Systolic Blood Pressure (SBP), WC, HC, WHR, WBC, ALT, AST, GLO, GGT, Urea, TG, LDL-C, and FBG (all *P* < 0.05). After adjusting for sex and age, MaR1 remained statistically positively associated with AST/ALT, ALB, A/G, and HDL-C (all *P* < 0.05) and negatively associated with BMI, WC, HC, WHR, ALT, GGT, UA, TG, and FBG (all *P* < 0.05).

**Table 3:** The correlations analysis of variables associated with serum Maresin-1 levels in study population.

	Serum Maresin-1		Serum Maresin-1 age- and sex-adjusted)	
	r	*P*-value	r	*P*-value
Sex	0.114	0.078	-	-
Age	-0.171	0.008	-	-
BMI	-0.331	0.000	-0.318	0.000
WC	-0.329	0.000	-0.294	0.000
HC	-0.204	0.001	-0.224	0.000
WHR	-0.360	0.000	-0.281	0.000
SBP	-0.211	0.001	-0.093	0.152
DBP	-0.113	0.081	-0.079	0.222
FBG	-0.285	0.000	-0.186	0.004
WBC	-0.155	0.016	-0.093	0.151
NEU	-0.060	0.352	-0.015	0.823
ALT	-0.289	0.000	-0.212	0.001
AST	-0.214	0.001	-0.102	0.116
AST/ALT	0.260	0.000	0.240	0.000
TP	0.016	0.810	0.006	0.931
ALB	0.157	0.015	0.172	0.008
GLO	-0.150	0.020	-0.123	0.058
A/G	0.204	0.002	0.156	0.016
GGT	-0.358	0.000	-0.158	0.015
ALP	-0.119	0.065	-0.085	0.193
Urea	-0.211	0.001	-0.125	0.055
UA	-0.126	0.051	-0.129	0.048
Crea	-0.068	0.294	-0.038	0.559
TC	-0.089	0.169	-0.072	0.270
TG	-0.330	0.000	-0.192	0.003
HDL-C	0.255	0.000	0.242	0.000
LDL-C	-0.150	0.020	-0.121	0.062
HCY	0.002	0.977	-0.013	0.837

Furthermore, binary logistic regression analysis was performed to investigate the association of MaR1 with NAFLD. When no adjustment was applied, serum MaR1 levels were significantly and inversely associated with the prevalence of NAFLD [odds ratio (OR) = 0.945; 95% confidence interval (CI) = 0.922–0.968, *P* = 0.000]. After adjusting for sex, age, BMI, BP, WHR, and FBG or further adjusting for ALT, AST, AST/ALT and UA, the association between circulating MaR1 levels and the presence of NAFLD was not affected [OR = 0.958, 95% CI = 0.928–0.989, *P* = 0.008] or [OR = 0.957, 95% CI = 0.926–0.989, *P* = 0.008]. Finally, even after adjusting for lipid profiles, MaR1 levels were significantly associated with NAFLD [OR = 0.965, 95% CI = 0.933–0.999, *P* = 0.042].

## Discussion

MaR1 has been identified as a new anti-inflammatory pro-resolving mediator with a vital role in metabolic disorders, including NAFLD [16–18, 23]. However, limited information supports the role of MaR1 in the occurrence and development of human NAFLD. In this cross-sectional study, serum MaR1 levels in NAFLD patients were significantly decreased compared with non-NAFLD subjects. Participants with the highest serum MaR1 quartile had a significantly lower prevalence of NAFLD than participants with the lowest serum MaR1 quartile. In addition, this study demonstrated that serum MaR1 concentrations were positively correlated with AST/ALT, ALB, A/G, and HDL-C and negatively correlated with age, obesity, FBG, ALT, GGT, UA, and TG. Importantly, the result showed that serum MaR1 levels were independently associated with NAFLD after adjusting for other potential confounders. Overall, these data indicate that MaR1 is an independent protective factor in NAFLD, and decrease in MaR1 levels may play a vital role in the pathophysiological process of NAFLD.

MaR1 was the first member of the maresin family to be identified. The biosynthesis of maresins mainly occurs in M2 macrophages and is initiated by the key enzyme 12-lipoxygenase (12-LOX) [24, 25]. DHA in macrophages is converted to 13S,14S-epoxy-maresin under the action of 12-LOX. Next, the epoxide intermediate forms the final product 7R, 14S-dihydroxydocosa-4Z, 8E, 10E, 12Z, 16Z, 19Z-hexaenoic acid (MaR1) through an epoxide-hydrolysis reaction [26]. In NAFLD, activated Kupffer cells release pro-inflammatory cytokines and chemokines [27], which promote the accumulation of pro-inflammatory M1-polarized monocytes in liver tissue [28], which disrupts the M1/M2 balance, resulting in a decrease in the M2 pro-resolving phenotype in liver tissue. Han et al. found that MaR1, as an endogenous ligand of RORα, increased the M2 polarity of liver macrophages by enhancing the expression and transcriptional activity of RORα [18]. Collectively, MaR1 is synthesized in M2 macrophages and promotes macrophages to shift to an M2 pro-resolving phenotype. When NAFLD occurs, the above loop is destroyed, resulting in decreased MaR1 synthesis. The present study found that serum MaR1 levels in NAFLD patients were significantly decreased compared with those in non-NAFLD participants, and with increasing circulating levels of MaR1, the prevalence of NAFLD decreased. Binary logistic regression analyses revealed that the level of MaR1 was an independent predictor of NAFLD.

NAFLD is a well-known liver phenotype of metabolic disorders [29], and the current results are consistent with those of previous reports [30], which revealed that NAFLD patients had aberrant metabolic features. In the present study, the NAFLD patients had significantly higher BP, BMI, FBG, WHR, ALT, AST, GGT, ALP, Urea, UA, Crea, TC, LDL-C, TG, and HCY levels but lower AST/ALT ratios and HDL-C levels than the non-NAFLD subjects. In addition, the results also showed that serum MaR1 levels were negatively associated with BMI, WC, HC, WHR, FBG, ALT, GGT, UA, and TG and positively associated with AST/ALT, ALB, A/G, and HDL-C. Moreover, MaR1 improves insulin resistance (IR) and lipid disorders by stimulating insulin signalling [17, 31, 32]. Overall, these results suggest that serum MaR1 levels are negatively associated with metabolic disorders, indicating that a decrease in serum MaR1 may promote the occurrence of NAFLD by aggravating IR and lipid metabolic disorders.

Recently, Félix-Soriano et al. found that MaR1 was significantly reduced in adipose tissue in aged HFD-induced obese mice compared to young normal controls [33]. Markworth et al. also showed that aged mouse muscle displayed significantly lower MaR1 levels than the young mouse muscle [34]. These data suggested that ageing is associated with MaR1 deficiency. The results of this cross-sectional study also showed that serum MaR1 levels were significantly negatively correlated with age in all participants.

### Study strength and limitations

This study is the first to explore the relationship between serum MaR1 levels and NAFLD in humans. The findings indicated that MaR1 is an independent protective factor against NAFLD. The present study also has some limitations. First, the current cross-sectional study is unable to illustrate the causal relationship between the serum MaR1 concentration and NAFLD and needs to be complemented by prospective studies. Second, abdominal ultrasound was used to diagnose NAFLD instead of liver biopsy, which can lead to missed diagnosis or misdiagnosis. Although hepatic steatosis, as an early stage of NAFLD, can be reliably identified by non-invasive imaging tests, including ultrasound, advanced NAFLD, such as NASH and cirrhosis, can only be definitively diagnosed by liver biopsy [35]. Thus, the lack of liver biopsy may be a weakness of the current research. Third, a selection bias may exist. All the participants who were recruited from the physical examination center may have stronger health awareness than non-participants. Finally, the current study may underestimate the true association because serum MaR1 levels were detected only once by ELISA kits, which is prone to random measurement error. Additionally, other eicosanoids such as protectins and resolvins may have shared effects with MaR1 on NAFLD, so this needs to be investigated to reflect the real relationship between MaR1 and NAFLD.

## Conclusions

This study found that serum MaR1 concentrations were decreased in NAFLD patients and that increased serum MaR1 levels were associated with a lower prevalence of NAFLD. These findings highlight the importance of MaR1 in comprehensive risk assessments of NAFLD. In clinical practice, MaR1 may be used to predict and prevent NAFLD. To reduce the prevalence of NAFLD, pursuing a higher MaR1 level may be appropriate. In the future, prospective studies with more comprehensive data and larger sample sizes are needed to confirm the contribution of Maresin-1 to the development of NAFLD.

## Data Availability

The datasets used and/or analyzed during the current study are available from the corresponding author on reasonable request.

## Abbreviations

NAFLD: Non-alcoholic fatty liver disease
NAFL: Non-alcoholic fatty liver
NASH: Non-alcoholic steatohepatitis
BMI: Body mass index
WC: Waist circumference
HC: Hip circumference
WHR: Waist-to-hip ratio
BP: Blood pressure
SBP: Systolic blood pressure
FBG: Fasting blood glucose
WBC: White blood cells
NEU: Neutrophil
ALT: Alanine aminotransferase
AST: Aspartate aminotransferase
TP: Total protein
ALB: Albumin
GLO: Globulin
A/G: Albumin-globulin-ratio
GGT: gamma-glutamyl transpeptidase
ALP: Alkaline phosphatase
UA: Uric acid
Crea: Creatinine
TC: Total cholesterol
TG: Triglyceride
HDL-C: High-density lipoprotein cholesterol
LDL-C: Low-density lipoprotein cholesterol
HCY: Homocysteine
